# Association of peripheral blood LUBAC and OTULIN expression with severity and outcome in acute ischemic stroke: a prospective cohort study

**DOI:** 10.3389/fimmu.2026.1792936

**Published:** 2026-05-20

**Authors:** Wei Chen, Zewen Chen, Hongqun Chen, Jun Li, Jiayan Xie, Xinyao Chen, Hongbei Xu

**Affiliations:** 1 Department of Neurology, The Affiliated Hospital of Guizhou Medical University, Guizhou, China; 2 Department of Basic Medicine, Guizhou Medical University, Guizhou, China

**Keywords:** acute ischemic stroke, functional outcome, LUBAC, OTULIN, severity

## Abstract

**Background:**

This study investigated whether peripheral blood expression of linear ubiquitin chain assembly complex (LUBAC) and OTU deubiquitinase with linear linkage specificity (OTULIN) is associated with stroke severity and functional outcome in acute ischemic stroke (AIS) patients.

**Methods:**

Immunofluorescence for LUBAC and OTULIN was performed in cortical autopsy specimens from two AIS cases. A total of 100 AIS patients and 100 age- and sex-matched healthy controls were enrolled. Peripheral blood mRNA levels of OTULIN and LUBAC components, including HOIL-1 interacting protein (HOIP), heme-oxidized IRP2 ubiquitin ligase 1L (HOIL-1L), and SHANK-associated RH domain interactor (SHARPIN), were quantified using quantitative reverse transcription polymerase chain reaction (qRT-PCR). Stroke severity at admission was assessed using the National Institutes of Health Stroke Scale (NIHSS). Functional outcome was evaluated using the modified Rankin Scale (mRS) at 90 ± 7 days. Regression models were used to evaluate associations of HOIP and OTULIN with stroke severity and outcome after adjustment for confounders. A HOIP × OTULIN interaction term was applied to assess effect modification. Discrimination for unfavorable outcome (mRS >2) was assessed using receiver operating characteristic (ROC) analysis.

**Results:**

Immunofluorescence suggested increased LUBAC and OTULIN expression in the peri-ischemic cortex. Peripheral blood HOIP and OTULIN expression levels were significantly higher in AIS patients than in healthy controls (P < 0.001). After adjustment for potential confounders, HOIP was independently positively associated with stroke severity (β = 1.928, P < 0.001) and independently associated with poor outcome (OR = 5.360, P = 0.013). OTULIN was just independently negatively associated with stroke severity (β = -1.060, P < 0.001), but its independent association with poor outcome was not statistically significant (P = 0.119). Interaction analysis showed a significant interaction between HOIP and OTULIN on stroke severity. ROC analysis showed that HOIP had good discriminative ability for poor outcome (AUC = 0.832). Adding HOIP to the clinical model increased the AUC from 0.846 to 0.907 and further improved model calibration and clinical net benefit.

**Conclusion:**

Peripheral blood HOIP and OTULIN may serve as candidate biomarkers associated with stroke severity in AIS, while HOIP may provide additional prognostic information for functional outcome.

## 1 Introduction

Stroke remains one of the leading causes of death and long-term disability worldwide and is a major contributor to the global burden of disease (1). The World Stroke Organization/Lancet Neurology Commission on Stroke 2 projects a 50% increase in global stroke mortality by 2050, underscoring a sustained and escalating worldwide stroke burden (2). Acute ischemic stroke (AIS) accounts for 69.6% - 72.8% of all stroke subtypes (3). Intravenous thrombolysis and endovascular mechanical thrombectomy are the principal acute reperfusion strategies for AIS (4). Nonetheless, neurological recovery remains limited because of the narrow treatment window, reperfusion injury, and substantial interindividual heterogeneity (5, 6). Accordingly, elucidating key pathological mechanisms and identifying accessible biomarkers remain major priorities for improving prognosis in AIS.

The pathophysiological cascade of AIS is triggered by an abrupt reduction in cerebral blood flow, leading to energy failure and subsequent tissue injury. Accumulating evidence indicates that sterile neuroinflammation is a major driver of the initiation, amplification, and persistence of ischemic damage (7). Post-stroke inflammation is not confined to the central nervous system (CNS) and can also trigger systemic immune activation (8). Following ischemic injury, neurons release damage-associated molecular patterns (DAMPs) that rapidly activate microglia and other innate immune cells within the CNS (9), while concurrently mobilizing peripheral immune responses (10). DAMP-driven signaling exacerbates blood-brain barrier (BBB) disruption and promotes recruitment of circulating immune cells into ischemic tissue, thereby intensifying neuroinflammation and neuronal injury (8, 10). Neutrophils, monocytes, and T and B lymphocytes contribute at distinct stages, and their abundance and activation states reflect the evolving pathological course of stroke (10). Clinically, multiple peripheral inflammatory markers, such as interleukin-1 beta, tumor necrosis factor alpha (TNF-α), interleukin 6 (IL-6), matrix metalloproteinase 9, C-reactive protein (CRP), the neutrophil-to-lymphocyte ratio (NLR), and the systemic immune-inflammation index (SII), have been associated with infarct volume, neurological deficit, and functional outcome (11, 12). These observations support the peripheral inflammatory milieu as an informative biological window for assessing AIS severity and prognosis.

Protein post-translational modification is a critical regulatory mechanism for neuroinflammation (13, 14). Among these, ubiquitination, particularly linear ubiquitination (M1-linked ubiquitin, M1-Ub), has emerged as an important mechanism modulating inflammatory response. The M1-Ub chain is specifically synthesized by the Linear Ubiquitin Chain Assembly Complex (LUBAC), a heterotrimer composed of HOIL-1-interacting protein (HOIP), heme-oxidized IRP2 ubiquitin ligase 1L (HOIL-1L), and SHANK-associated RH domain interactor (SHARPIN) (15, 16). HOIP serves as the catalytic core and stabilizes the complex through interaction with HOIL-1L and SHARPIN via its ubiquitin-like domain (17). As the only E3 ligase complex known to catalyze M1-Ub chain formation, LUBAC is essential for immune homeostasis, regulation of nuclear factor kappa B (NF-κB) signaling, and cell survival programs (18–21). The abundance of M1-Ub chains is counterbalanced by deubiquitinases, notably OTU deubiquitinase with linear linkage specificity (OTULIN) and cylindromatosis (CYLD), with OTULIN exhibiting substantially higher catalytic efficiency than CYLD (16). Animal and clinical studies have demonstrated that dysregulation of LUBAC and OTULIN can cause peripheral immune abnormalities, such as early-onset systemic autoinflammatory syndrome caused by OTULIN deficiency (22). In inflammatory bowel disease models, macrophage-specific LUBAC deficiency suppresses NF-κB and extracellular signal-regulated kinase signaling and attenuates inflammatory response (23). In addition, mutation in HOIL-1L has been linked to immunodeficiency accompanied by excessive inflammatory responses (24). Beyond systemic immunity, linear ubiquitination has also been implicated in misfolded protein handling and neuroinflammatory regulation across several CNS disorders, including Alzheimer disease, amyotrophic lateral sclerosis, and Huntington disease (25–27).

Although LUBAC and OTULIN are recognized regulators of immune signaling, their relevance to AIS remains unclear. Our preliminary work demonstrated that experimental ischemic stroke in rats increased microglial OTULIN expression, and that OTULIN overexpression attenuated neurological deficits, reduced infarct volume, and alleviated neuroinflammatory response. These effects were accompanied by inhibition of NF-κB signaling (28, 29). However, the peripheral blood expression profiles of LUBAC and OTULIN in AIS, and their associations with disease severity and outcome have not been systematically explored. The present study therefore characterized peripheral blood LUBAC and OTULIN expression in AIS and evaluated their clinical significance, providing evidence for the role of linear ubiquitination in AIS patients.

## 2 Materials and methods

### 2.1 Study participants

This study was conducted in the Department of Neurology, Guiyang Second People’s Hospital, between November 2024 and August 2025. Patients with AIS admitted within 48 hours of symptom onset were screened for eligibility. Inclusion criteria were as follows: (1) AIS diagnosed according to the 2023 Chinese Guidelines for Ischemic Stroke Diagnosis and Treatment and confirmed by head CT or MRI (3); (2) age 18–80 years; (3) ability to cooperate with study assessments; and (4) willingness to provide peripheral blood samples. Exclusion criteria were as follows: (1) intracerebral hemorrhage or transient ischemic attack; (2) severe infection, malignancy, or immunodeficiency; (3) use of immunosuppressive agents; (4) aphasia, hearing impairment, or severe disability that precluded participation in study assessments; and (5) life expectancy < 3 months. Stroke etiology was classified according to the Trial of ORG 10172 in Acute Stroke Treatment (TOAST) criteria (30). Age- and sex-matched healthy controls were recruited from the hospital’s health examination center. The study protocol was approved by the Ethics Committee of Guiyang Second People’s Hospital (JYYY-2024-WZ-26), and written informed consent was obtained from all participants.

### 2.2 Baseline data collection

On admission, demographic and clinical data were collected, including age, sex, body mass index (BMI), hypertension, diabetes, smoking history, alcohol consumption, coronary artery disease, atrial fibrillation, and a history of previous strokes. Stroke severity and functional outcome were assessed using the National Institutes of Health Stroke Scale (NIHSS) and the modified Rankin Scale (mRS). Laboratory parameters included white blood cell count, neutrophil count, lymphocyte count, platelet count, fasting blood glucose, total cholesterol, triglycerides, HDL-C, LDL-C, homocysteine, C-reactive protein, IL-6, D-dimer, and coagulation parameters. Based on peripheral blood cell parameters, NLR and SII were calculated.

### 2.3 Sample collection and quantitative real-time PCR for LUBAC and OTULIN mRNA

Peripheral blood was collected in EDTA anticoagulant tubes in the morning after an overnight fast, and immediately stabilized with RNALater™ RNA stabilization solution (Beyotime, China, Cat. R0116). Total RNA was extracted using the RNAeasy™ Blood RNA Extraction Kit (Beyotime, China, Cat. R0091M). RNA concentration and purity were assessed using a Nanodrop ND-1000 spectrophotometer. cDNA was synthesized using the Evo M-MLV RT Mix Kit with gDNA Clean (Accurate Biology, China, Cat. AG11728). qRT-PCR was performed using a reaction mixture with SYBR Green chemistry on a CFX™ Systems (Bio-Rad, USA). Relative mRNA expression levels were calculated using the 2^^-△△Ct^ method. Primer sequences were as follows: GAPDH forward 5′-GTCTCCTCTGACTTCAACAGCGG-3′ and reverse 5′-ACACCAGTGGATGCAGGTT-3′; HOIP forward 5′-CTGGATCTGTCATGGCAACCTTG-3′ and reverse 5′-ACATCACCTCCGTGTCGTCGAACA-3′; HOIL-1L forward 5′-TGACAACCACTACTCGTGTCGTCG-3′ and reverse 5′-CACTGGGGTGTTTTGCGAATGGAG-3′; SHARPIN forward 5′-TGGCATGGATGTGTAGGACC-3′ and reverse 5′-ACTGGACGAGTGGAGTGTGGAAG-3′; OTULIN forward 5′-GACAGCTTCTGAGGAACACCT-3′ and reverse 5′-TCCGTGTTGTTACTTGGAGAGCC-3′. All assays were performed independently by two investigators.

### 2.4 Stroke severity and functional outcome assessment

Stroke severity was evaluated using the NIHSS score at admission. Patients were categorized into four groups: mild (0-4), moderate (5-15), moderate to severe (16-20), and severe (≥20) (31). Functional outcome was assessed by outpatient visit or telephone follow-up at 90 ± 7 days after stroke onset. Outcomes were dichotomized as favorable (mRS ≤2) and unfavorable (mRS >2) (32).

### 2.5 Postmortem tissue immunofluorescence

Postmortem brain tissues were obtained from AIS patients at Guiyang Second People’s Hospital, with written informed consent from the deceased’s legally authorized representative. The clinical characteristics and relevant radiological information for the two cases are provided in **Supplementary Table 1** and **Supplementary Figure 1**. Samples were collected from the peri-ischemic cortex and the contralateral non-ischemic cortex, fixed in 10% buffered formalin for 4 weeks, and embedded in paraffin. The brain sections (8 μm) were deparaffinized in xylene, rehydrated through graded ethanol, and washed in PBS. Antigen retrieval was performed in EDTA buffer (pH 8.0). Sections were blocked with 5% normal donkey serum at 37 °C for 30 min and incubated overnight at 4 °C with one of the following primary antibodies: HOIP (DF13379, Affinity Biosciences, USA), HOIL-1L (bs-19761R, Bioss, China), SHARPIN (14626-1-AP, Proteintech, China), or OTULIN (bs-14689R, Bioss, China). Sections were then incubated with CoraLite594-labeled donkey anti-rabbit IgG (SA00013-8, Proteintech, China)) for 1 hour at room temperature and counterstained with DAPI (Sigma, USA). All images were captured using an A1R laser confocal microscope (Nikon, Tokyo, Japan) under identical acquisition settings.

### 2.6 Statistical analysis

All analyses were performed using R (version 4.4.1) and GraphPad Prism 10. Continuous variables were presented as mean ± standard deviation (SD) or median (interquartile range, IQR), and categorical variables as frequency (%). Normality was assessed using the Shapiro-Wilk test, and homogeneity of variance was tested using the F-test. For group comparison, continuous variables were analyzed using t-test or Mann-Whitney U test, and categorical variables were analyzed using χ² test or Fisher’s exact test.

To evaluate the associations of peripheral blood HOIP and OTULIN expression with stroke severity, admission NIHSS score was analyzed as a continuous outcome using linear regression. Univariable and multivariable analyses were performed. Three stepwise multivariable models were constructed: Model 1 included HOIP, OTULIN, age, and sex; Model 2 further adjusted for smoking, hypertension, and diabetes mellitus; and Model 3 additionally adjusted for TOAST classification, with other determined etiology and undetermined etiology combined because of small sample sizes. Model assumptions, including residual normality, homoscedasticity, multicollinearity, and influential observations, were assessed, and Heteroskedasticity-Consistent 3 (HC3) robust standard errors were applied when appropriate. As a sensitivity analysis, ordinal logistic regression based on NIHSS severity categories was also performed using the same adjustment strategy, and the proportional odds assumption was evaluated with the Brant test. For functional outcome analysis, 90-day outcome was dichotomized as favorable (mRS ≤2) or poor (mRS >2) and analyzed using logistic regression. Univariable and multivariable analyses were conducted. Three stepwise multivariable models were constructed: Model 1 included HOIP, OTULIN, age, and sex; Model 2 further adjusted for smoking, hypertension, and diabetes mellitus; and Model 3 additionally adjusted for admission NIHSS score.

To assess the interaction between HOIP and OTULIN, regression models incorporating the main effects of HOIP and OTULIN and their interaction term (HOIP × OTULIN) were constructed, with NIHSS score and 90 ± 7-day functional outcome analyzed separately as dependent variables (33–35). For NIHSS score, linear regression was used, and the residual distribution and homoscedasticity of the final model were examined to assess model fit. Because NIHSS is an ordinal clinical scale, ordinal logistic regression was additionally performed as a sensitivity analysis to evaluate the robustness of the results. Three stepwise adjustment models were fitted: Model 1 included HOIP, OTULIN, and their interaction term; Model 2 further adjusted for age and sex; and Model 3 further adjusted for smoking, hypertension, diabetes mellitus, and TOAST classification. For functional outcome, logistic regression was performed using the same interaction framework. Three stepwise adjustment models were fitted: Model 1 included HOIP, OTULIN, and their interaction term; Model 2 further adjusted for age and sex; and Model 3 further adjusted for smoking, hypertension, diabetes mellitus, and admission NIHSS score. For models in which the interaction term was statistically significant, simple slope analyses were conducted to estimate the marginal effect of OTULIN at the 25th, 50th, and 75th percentiles of HOIP.

In addition, the discriminatory ability of HOIP and OTULIN for 90-day poor functional outcome was assessed using receiver operating characteristic (ROC) curves, with the area under the curve (AUC) calculated (36). To evaluate the incremental prognostic value of HOIP, three logistic regression models were constructed: Model 1 included age and sex; Model 2 further included admission NIHSS score; and Model 3 additionally included HOIP. Calibration curves were used to assess agreement between predicted and observed probabilities (37), and decision curve analysis (DCA) was performed to evaluate potential clinical utility (38). Internal validation was performed using 1,000 bootstrap resamples (39). All tests were two-tailed, with P < 0.05 considered statistically significant.

## 3 Results

### 3.1 Expression of LUBAC and OTULIN in patients with AIS

**Figure 1:**
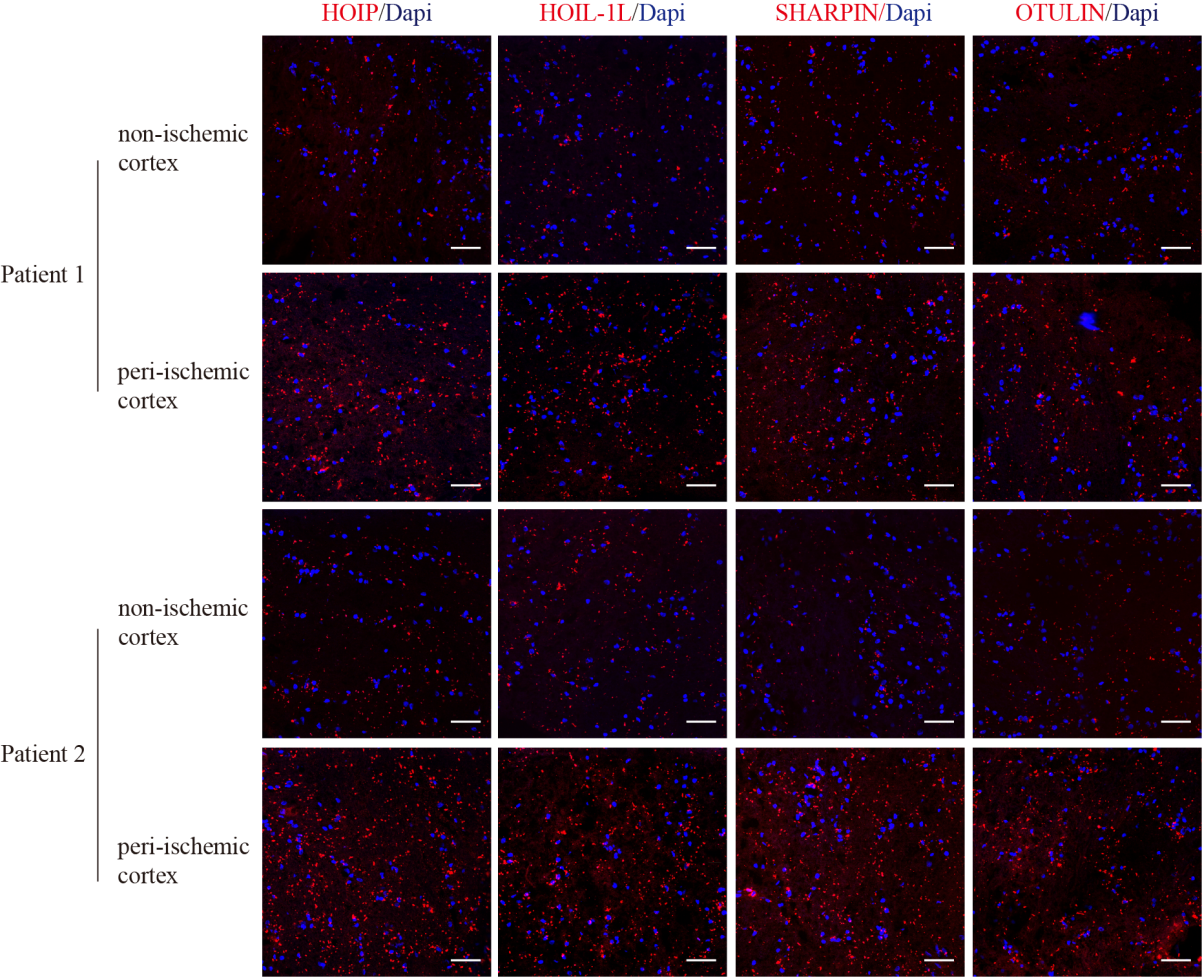
Representative images of immunofluorescence of LUBAC and OTULIN in AIS patients. Scale bar = 20 μm.

Immunofluorescence staining for the LUBAC components and OTULIN suggested higher expression in the peri-ischemic cortex than in the non-ischemic cortex in both Patient 1 and Patient 2 (**Figure 1**). Because only two autopsy cases were available, no statistical analysis was performed, and these findings are presented as descriptive observation.

### 3.2 Baseline characteristics

The baseline clinical characteristics are shown in **Table 1**. No significant differences were observed between the AIS and control groups in terms of age, sex, smoking, alcohol consumption, coronary artery disease, and atrial fibrillation (p > 0.05). AIS patients had significantly higher rates of hypertension, diabetes, hyperlipidemia, BMI, triglycerides, fasting blood glucose, homocysteine, and IL-6l levels than the healthy controls. Moreover, HOIP and OTULIN mRNA expression were significantly increased in the AIS group (HOIP: 3.22 vs. 1.29; OTULIN: 3.61 vs. 1.06; both P < 0.001), whereas SHARPIN and HOIL-1L expression levels did not differ between groups (P > 0.05).

**Table 1:** Baseline demographics and clinical characteristics of this study.

Item	Overall	AIS	Control	P value
N	200	100	100	
Age, mean ± SD	62.84 (9.45)	63.99 (9.08)	61.69 (9.72)	0.085
Male (%)	109 (54.5)	58 (58.0)	51 (51.0)	0.394
Smoking (%)	82 (41.0)	48 (48.0)	34 (34.0)	0.062
Alcohol (%)	83 (41.5)	44 (44.0)	39 (39.0)	0.566
BMI (kg/m^2^), mean ± SD	25.04 (4.42)	25.82 (4.58)	24.26 (4.13)	0.012
Hypertension (%)	65 (32.5)	40 (40.0)	25 (25.0)	0.035
Hyperlipidemia (%)	58 (29.0)	36 (36.0)	22 (22.0)	0.043
Diabetes mellitus (%)	60 (30.0)	39 (39.0)	21 (21.0)	0.009
Coronary heart disease (%)	43 (21.5)	25 (25.0)	18 (18.0)	0.302
Atrial fibrillation (%)	17 (8.5)	12 (12.0)	5 (5.0)	0.128
Recurrent stroke (%)	32 (16.0)	32 (32.0)	-	-
Admission NIHSS score, median (IQR)	6.00 (5.00, 9.00)	6.00 (5.00, 9.00)	-	-
90 ± 7d (mRS), median (IQR)	2.00 (2.00, 3.00)	2.00 (2.00, 3.00)	-	-
TOAST classification of stroke etiology (%)				
large artery atherosclerosis	47 (23.5)	47 (47.0)	-	-
Cardioembolic stroke	16 (8.0)	16 (16.0)	-	-
Small artery occlusion	26 (13.0)	26 (26.0)	-	-
Other determined cause	7 (3.5)	7 (7.0)	-	-
Undetermined cause	4 (2.0)	4 (4.0)	-	-
Stroke treatment(%)				
Antiplatelet therapy	52 (26.0)	52 (52.0)	-	-
Anticoagulant therapy	16 (8.0)	16 (16.0)	-	-
Intravenous thrombolysis + endovascular therapy	12 (6.0)	12 (12.0)	-	-
Intravenous thrombolysis	11 (5.5)	11 (11.0)	-	-
Endovascular therapy	9 (4.5)	9 (9.0)	-	-
Onset-admission time (h), median (IQR)	12.00 (4.38, 22.25)	12.00 (4.38, 22.25)	-	-
Hospitalization duration (d), median (IQR)	15.00 (10.00, 20.25)	15.00 (10.00, 20.25)	-	-
White blood cell (10^9^/L), median (IQR)	7.56 (1.70)	7.49 (1.92)	7.64 (1.46)	0.532
Monocyte count (10^9^/L), median (IQR)	0.49 (0.38, 0.58)	0.50 (0.32, 0.58)	0.48 (0.42, 0.59)	0.196
Neutrophil count (10^9^/L), median (IQR)	4.90 (3.08, 5.96)	4.68 (2.92, 5.92)	5.06 (3.79, 5.96)	0.292
Lymphocyte count (10^9^/L), median (IQR)	1.92 (1.41, 2.38)	2.04 (1.52, 2.41)	1.90 (1.39, 2.33)	0.312
Platelet coun (10^9^/L), median (IQR)	236.00 (201.00, 276.25)	240.50 (208.75, 267.50)	230.50 (185.75, 296.50)	0.752
CRP (mg/L), median (IQR)	3.60 (2.34, 6.12)	4.20 (2.00, 7.65)	3.30 (2.46, 5.40)	0.089
IL-6 (pg/mL), median (IQR)	4.38 (2.73, 5.64)	5.62 (3.89, 10.70)	3.49 (2.21, 4.56)	<0.001
NLR, median (IQR)	2.61 (1.41, 3.88)	2.37 (1.35, 3.66)	2.72 (1.77, 4.21)	0.151
SII, median (IQR)	572.58 (353.79, 890.52)	534.82 (340.94, 820.71)	583.06 (362.50, 959.11)	0.227
Fasting blood glucose (mmol/L), median (IQR)	6.52 (5.57, 7.30)	6.87 (6.42, 8.30)	5.57 (4.99, 6.62)	<0.001
Triglycerides (mmol/L), median (IQR)	1.16 (1.06, 1.45)	1.19 (1.09, 1.37)	1.15 (1.02, 1.46)	0.041
Total cholesterol (mmol/L), median (IQR)	4.31 (3.54, 4.78)	4.28 (3.50, 4.91)	4.33 (3.56, 4.72)	0.928
LDL-C (mmol/L), mean ± SD	2.57 (0.81)	2.57 (0.89)	2.58 (0.72)	0.945
HDL-C (mmol/L), median (IQR)	1.21 (1.10, 1.41)	1.23 (1.16, 1.36)	1.17 (0.93, 1.51)	0.099
PT (s), mean ± SD	12.27 (1.57)	12.49 (1.50)	12.05 (1.62)	0.051
APTT (s), median (IQR)	29.40 (27.20, 31.52)	29.46 (27.74, 31.01)	29.16 (26.28, 32.67)	0.686
Fibrinogen (g/L), median (IQR	3.34 (3.01, 3.80)	3.34 (3.06, 3.68)	3.37 (2.83, 4.03)	0.898
D-dimer (ug/mL), median (IQR)	0.84 (0.72, 1.35)	0.84 (0.78, 1.25)	0.83 (0.62, 1.41)	0.091
Homocysteine (umol/L), median (IQR)	11.59 (10.75, 13.91)	13.57 (11.43, 17.98)	10.74 (9.81, 12.06)	<0.001
HOIP, median (IQR)	1.70 (1.23, 3.21)	3.22 (2.50, 3.79)	1.29 (1.07, 1.50)	<0.001
HOIL-1L, mean ± SD	1.21 (0.45)	1.26 (0.42)	1.17 (0.48)	0.701
OTULIN, median (IQR)	1.38 (1.00, 3.59)	3.61 (1.78, 5.35)	1.06 (0.79, 1.25)	<0.001
SHARPIN, median (IQR)	1.29 (1.14, 1.48)	1.33 (1.18, 1.48)	1.26 (1.10, 1.47)	0.102

AIS, acute ischemic stroke; APTT (or aPTT), activated partial thromboplastin time; CRP, C-reactive protein; d, day; h, hour; HDL-C, high-density lipoprotein cholesterol; LDL-C, low-density lipoprotein cholesterol; NLR, neutrophil-to-lymphocyte ratio; PT, prothrombin time; SII, systemic immune-inflammation index. Continuous variables are presented as mean (SD), categorical variables as frequency (%). Comparisons between groups were performed using t-tests or Chi-square tests.

### 3.3 Association of HOIP and OTULIN with stroke severity, functional outcome, and inflammatory markers

**Figure 2:**
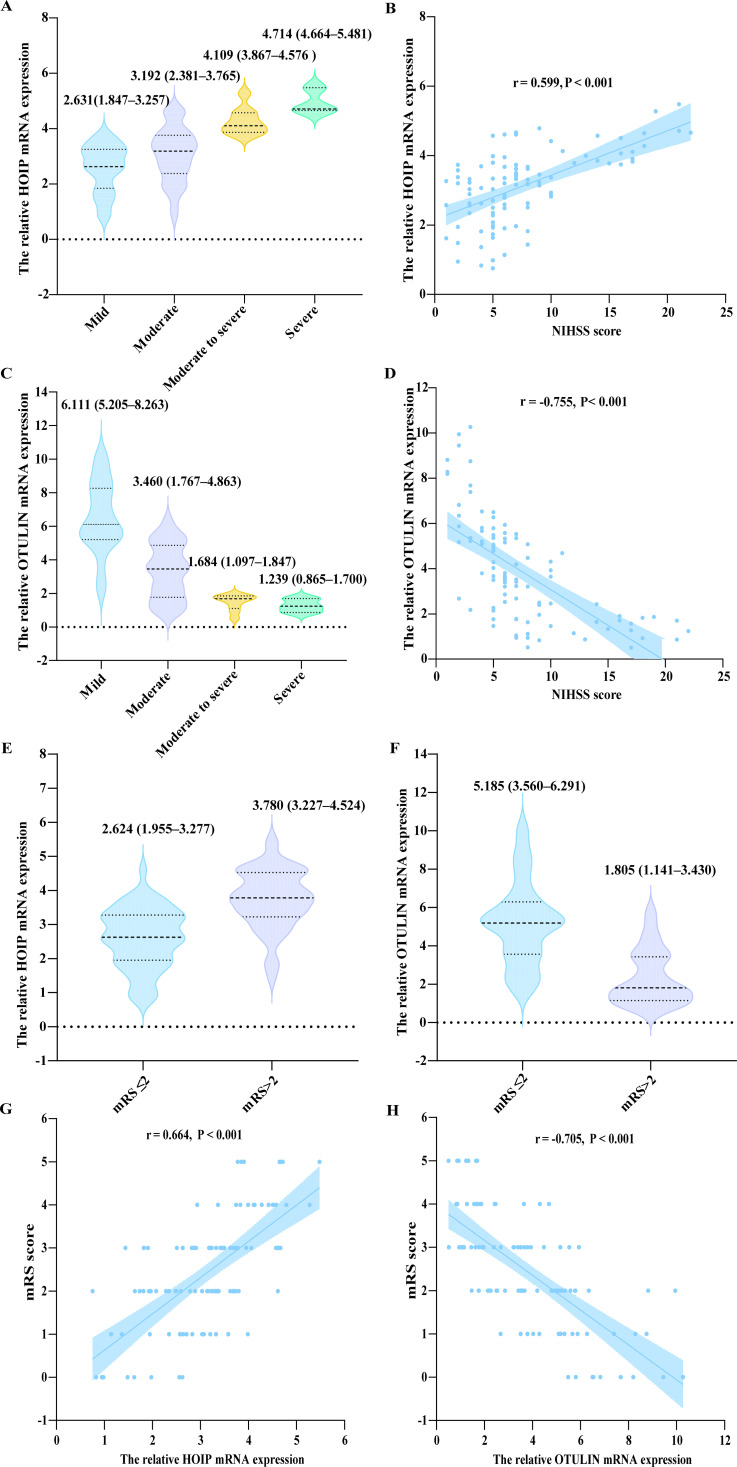
Elevated expression levels of HOIP and OTULIN mRNA are associated with stroke severity and functional outcome in patients with AIS. **(A–D)** HOIP and OTULIN mRNA expression across NIHSS severity groups and their correlation with NIHSS score. **(E–H)** HOIP and OTULIN mRNA expression by mRS outcome (mRS ≤2 vs mRS>2) and their correlation with mRS score.

**Figure 3:**
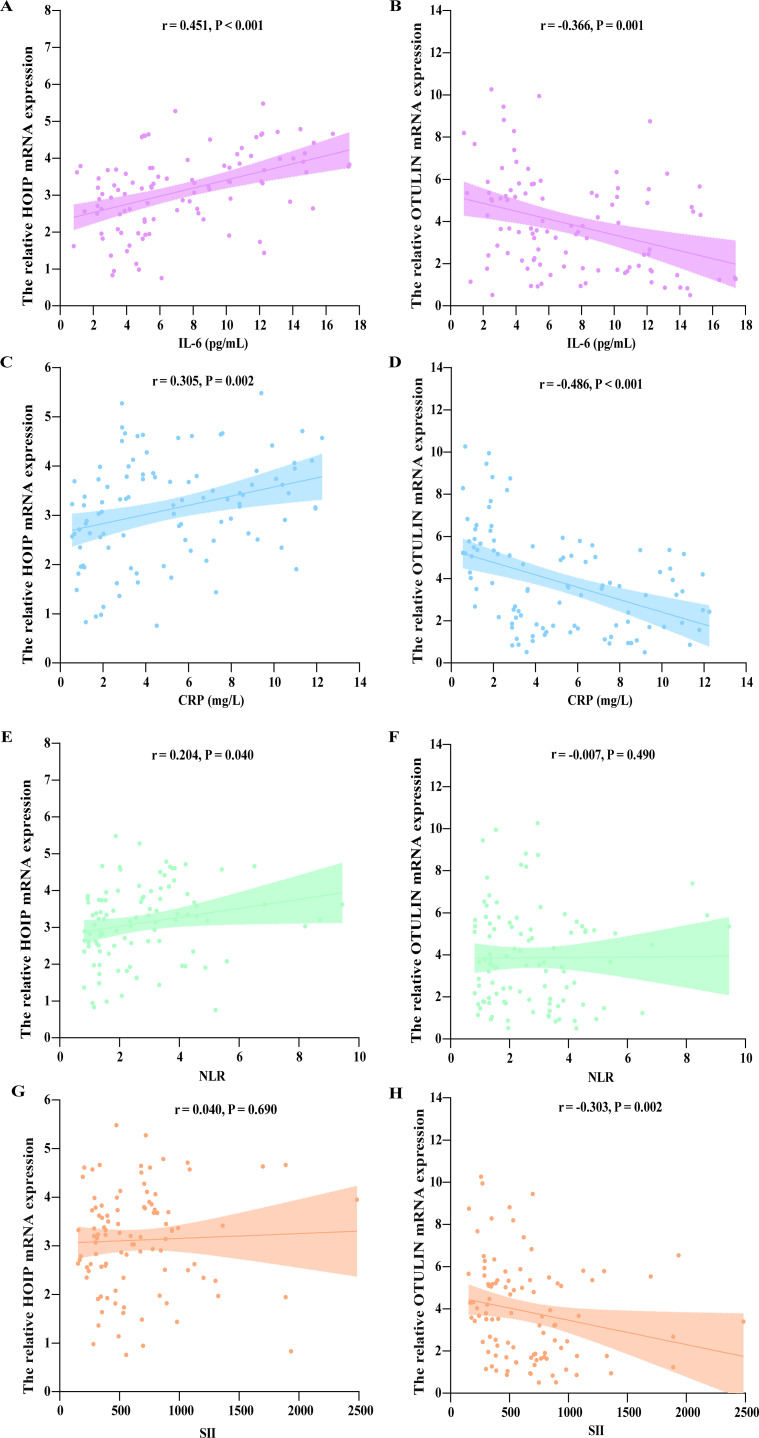
Correlation of HOIP and OTULIN mRNA expression with inflammatory markers in patients with AIS. **(A, B)** HOIP and OTULIN mRNA expression in relation to IL-6 level. **(C, D)** HOIP and OTULIN mRNA expression in relation to CRP levels. **(E, F)** HOIP mRNA expression in relation to NLR and SII. **(G, H)** OTULIN mRNA expression in relation to NLR and SII.

For AIS patients, HOIP expression was positively correlated with NIHSS score (r = 0.599, P < 0.001; **Figures 2A, B**), whereas OTULIN expression was inversely correlated with NIHSS (r = −0.755, P < 0.001; **Figures 2C, D**). Regarding functional outcome, HOIP expression was higher in the poor outcome group, while OTULIN expression was lower (**Figures 2E, F**). Consistently, HOIP correlated positively with mRS, whereas OTULIN correlated negatively with mRS (P < 0.001, **Figures 2G, H**). For inflammatory markers, OTULIN showed inverse correlations with IL-6, CRP, and SII, whereas HOIP showed positive correlations with IL-6, CRP, and NLR (P < 0.05, **Figures 3A–H**).

In univariable linear regression analysis, HOIP was positively associated with NIHSS score (β = 2.801, P < 0.001) whereas OTULIN was inversely associated with NIHSS (β = −1.402, P < 0.001) (**Table 2**). In multivariable linear regression, higher HOIP expression remained independently associated with higher NIHSS score (β = 1.928, 95% CI 1.263-2.594; P < 0.001), whereas higher OTULIN expression remained independently associated with lower NIHSS score (β = -1.060, 95% CI -1.364 to -0.755; P < 0.001) (**Table 3**). Model diagnostics showed acceptable residual normality (Shapiro–Wilk test, P = 0.087) and no substantial multicollinearity, although mild heteroscedasticity was detected (P = 0.022). After applying HC3 robust standard errors, the direction and statistical significance of the associations between HOIP, OTULIN, and NIHSS score remained unchanged. Ordinal logistic regression based on NIHSS severity categories yielded directionally consistent results, and Brant tests indicated no violation of the proportional odds assumption (**Supplementary Table 2**). For poor functional outcome, HOIP remained independently associated with poor outcome in the fully adjusted logistic regression model (OR = 5.360, 95% CI 1.421-20.228; P = 0.013), whereas OTULIN was not independently associated with poor outcome (OR = 0.588, 95% CI 0.302-1.145; P = 0.119) (**Table 3**).

**Table 2:** Univariable analysis for stroke severity and poor outcome.

Variable	NIHSS score (β, 95% CI)	P value	mRS >2 (OR, 95% CI)	P value
Age (years)	-0.022 (-0.129, 0.084)	0.684	0.998 (0.956, 1.043)	0.937
Sex (male)	-1.526 (-3.454, 0.404)	0.124	2.182 (0.971, 4.901)	0.059
Smoking	-1.324 (-3.235, 0.587)	0.178	2.233 (1.001, 4.982)	0.050
Alcohol	-0.330 (-2.270, 1.611)	0.740	1.333 (0.603, 2.948)	0.477
BMI	0.049 (-0.163, 0.260)	0.652	0.999 (0.916, 1.089)	0.980
Hypertension	1.242 (-0.710, 3.193)	0.215	0.790 (0.353, 1.769)	0.566
Hyperlipidemia	-1.097 (-3.093, 0.899)	0.284	1.286 (0.567, 2.916)	0.547
Diabetes mellitus	0.054 (-1.922, 2.030)	0.958	1.010 (0.451, 2.262)	0.980
Coronary heart disease	1.120 (-1.094, 3.334)	0.324	2.182 (0.840, 5.669)	0.109
Atrial fibrillation	-2.250 (-5.182, 0.682)	0.136	0.569 (0.167, 1.930)	0.365
Recurrent stroke	-0.246 (-2.312, 1.819)	0.815	1.143 (0.491, 2.661)	0.757
NIHSS score	-	-	3.688 (2.068, 6.575)	<0.001
mRS score	3.052 (2.643, 3.462)	<0.001		
TOAST classification of stroke	-0.882 (-1.687, -0.077)	0.034	0.803 (0.568, 1.135)	0.214
Stroke treatment	0.496 (-0.200, 1.192)	0.166	1.293 (0.964, 1.734)	0.087
Onset-admission time (h)	-0.002 (-0.095, 0.092)	0.972	1.001 (0.964, 1.040)	0.939
Hospitalization duration (d)	0.090 (-0.037, 0.216)	0.168	1.051 (0.995, 1.110)	0.074
White blood cell count	0.060 (-0.446, 0.566)	0.817	0.998 (0.812, 1.227)	0.988
Monocyte count	-0.007 (-5.556, 5.542)	0.998	2.595 (0.264, 25.493)	0.413
Neutrophil count	0.221 (-0.287, 0.729)	0.396	1.063 (0.863, 1.309)	0.567
Lymphocyte count	-1.599 (-3.132, -0.067)	0.044	0.522 (0.268, 1.017)	0.056
Platelet count	0.011 (-0.016, 0.039)	0.411	1.010 (0.998, 1.021)	0.101
CRP	0.671 (0.422, 0.919)	<0.001	1.492 (1.267, 1.757)	<0.001
IL-6	0.671 (0.489, 0.853)	<0.001	1.292 (1.148, 1.454)	<0.001
NLR	0.359 (-0.191, 0.909)	0.204	1.111 (0.883, 1.397)	0.368
SII	0.002 (-0.004, 0.004)	0.148	1.001 (1.000, 1.002)	0.261
Fasting blood glucose	0.178 (-0.621, 0.977)	0.664	1.097 (0.792, 1.521)	0.577
Triglycerides	0.385 (-4.267, 5.037)	0.872	0.874 (0.131, 5.850)	0.890
Total cholesterol	0.866 (-0.200, 1.932)	0.114	1.430 (0.903, 2.263)	0.127
LDL-C	0.041 (-1.047, 1.130)	0.941	0.949 (0.608, 1.480)	0.817
HDL-C	8.405 (0.715, 16.095)	0.034	1.355 (0.055, 33.582)	0.853
PT	0.102 (-0.544, 0.747)	0.758	1.037 (0.797, 1.351)	0.785
APTT	0.134 (-0.357, 0.624)	0.595	0.973 (0.796, 1.189)	0.789
Fibrinogen	1.102 (-1.512, 3.715)	0.41	1.714 (0.584, 5.032)	0.327
D-dimer	-2.242 (-5.596, 1.112)	0.193	0.713 (0.178, 2.853)	0.632
Homocysteine	-0.260 (-0.522, 0.002)	0.054	0.969 (0.869, 1.081)	0.574
HOIP	2.801 (2.059, 3.543)	<0.001	4.588 (2.418, 8.706)	<0.001
HOIL-1L	-1.028 (-4.531, 2.475)	0.567	0.933 (0.223, 3.909)	0.925
OTULIN	-1.402 (-1.713, -1.092)	<0.001	0.415 (0.298, 0.579)	<0.001
SHARPIN	0.825 (-1.474, 3.123)	0.484	1.991 (0.753, 5.269)	0.473

**Table 3:** Multivariable analysis for stroke severity and poor outcome.

Variable	NIHSS score (β, 95% CI)	P value	mRS >2 (OR, 95% CI)	P value
Model 1				
Age	-0.010 (-0.080, 0.060)	0.783	0.979 (0.912, 1.052)	0.567
Sex (male)	-0.036 (-1.334, 1.262)	0.956	1.168 (0.333, 4.094)	0.808
OTULIN	-1.089 (-1.383, -0.795)	<0.001	0.397 (0.258, 0.612)	<0.001
HOIP	1.959 (1.317, 2.602)	<0.001	5.015 (2.191, 11.478)	<0.001
Model 2				
Age	-0.009 (-0.081, 0.064)	0.817	0.995 (0.923, 1.072)	0.889
Sex (male)	0.009 (-1.419, 1.436)	0.990	0.798 (0.171, 3.721)	0.774
Smoking	-0.302 (-1.739, 1.134)	0.681	2.477 (0.515, 11.923)	0.258
Hypertension	0.235 (-1.069, 1.538)	0.725	1.257 (0.328, 4.808)	0.739
Diabetes mellitus	0.938 (-0.356, 2.233)	0.159	0.442 (0.116, 1.679)	0.230
OTULIN	-1.094 (-1.393, -0.795)	<0.001	0.400 (0.258, 0.618)	<0.001
HOIP	1.970 (1.323, 2.618)	<0.001	6.039 (2.405, 15.166)	<0.001
Model 3				
Age (years)	0.002 (-0.072, 0.076)	0.957	0.957 (0.862, 1.062)	0.409
Sex (male)	-0.070 (-1.494, 1.355)	0.924	0.167 (0.015, 1.893)	0.149
Smoking	0.184 (-1.264, 1.631)	0.804	1.800 (0.890, 5.574)	0.061
Hypertension	-0.323 (-1.636, 0.990)	0.631	0.846 (0.159, 4.508)	0.844
Diabetes mellitus	-0.867 (-2.163, 0.429)	0.193	0.606 (0.103, 3.567)	0.579
OTULIN	-1.060 (-1.364, -0.755)	<0.001	0.588 (0.302, 1.145)	0.119
HOIP	1.928 (1.263, 2.594)	<0.001	5.360 (1.421, 20.228)	0.013
CE vs LAA	-1.618 (-3.492, 0.256)	0.094	-	-
SAA vs LAA	-0.749 (-2.326, 0.828)	0.354	-	-
SOE/SUE vs LAA	-1.364 (-3.530, 0.801)	0.220	-	-
Admission NIHSS	-	-	3.250 (1.650, 6.405)	<0.001

CE, cardioembolic stroke; LAA, large artery atherosclerosis; SAA, small artery occlusion; SOE, stroke of other determined etiology; SUE, stroke of undetermined etiology. TOAST classification was used as a categorical variable in the regression model, with LAA as the reference category.

### 3.4 Interaction between HOIP and OTULIN

In multivariable linear regression model with NIHSS as outcome, the OTULIN × HOIP interaction term was consistently significant across the unadjusted model and progressively adjusted models (P < 0.001, **Table 4**). Simple slopes analyses (**Supplementary Table 3**) showed that the inverse association between OTULIN and NIHSS was stronger at higher HOIP levels. At the 25th, 50th, and 75th percentiles of HOIP, the β coefficients for OTULIN were -0.783, -1.244, and -1.611, respectively (P < 0.001). The direction and statistical significance of the associations for HOIP, OTULIN, and their interaction term were preserved after applying HC3 robust standard errors and after excluding potentially influential observations (**Supplementary Tables 4A, B**). Ordinal logistic regression using NIHSS severity categories also yielded directionally consistent results, and Brant tests showed no violation of the proportional odds assumption (**Supplementary Table 5**). However, in the logistic regression model for poor outcome, the interaction term did not reach statistical significance (**Table 4**).

**Table 4:** Interaction effects of OTULIN × HOIP for stroke severity and poor outcome.

Variable	NIHSS score (β, 95% CI)	P value	mRS >2 (OR, 95% CI)	P value
Model 1				
OTULIN	-1.207 (-1.462, -0.953)	<0.001	0.396 (0.247, 0.575)	< 0.001
HOIP	1.802 (1.241, 2.363)	<0.001	4.643 (2.106, 12.746)	< 0.001
OTULIN × HOIP	-0.641 (-0.869, -0.413)	<0.001	0.967 (0.607, 1.581)	0.888
Model 2				
OTULIN	-1.185 (-1.446, -0.924)	<0.001	0.398 (0.243, 0.584)	< 0.001
HOIP	1.812 (1.243, 2.380)	<0.001	4.856 (2.124, 13.902)	< 0.001
OTULIN × HOIP	-0.652 (-0.883, -0.421)	<0.001	0.965 (0.601, 1.591)	0.883
Model 3				
OTULIN	-1.182 (-1.455, -0.909)	<0.001	0.398 (0.156, 1.104)	0.129
HOIP	1.789 (1.198, 2.380)	<0.001	5.034 (1.634, 14.709)	0.004
OTULIN × HOIP	-0.640 (-0.877, -0.403)	<0.001	1.116 (0.563, 2.468)	0.766

### 3.5 Prognostic value of HOIP

**Figure 4:**
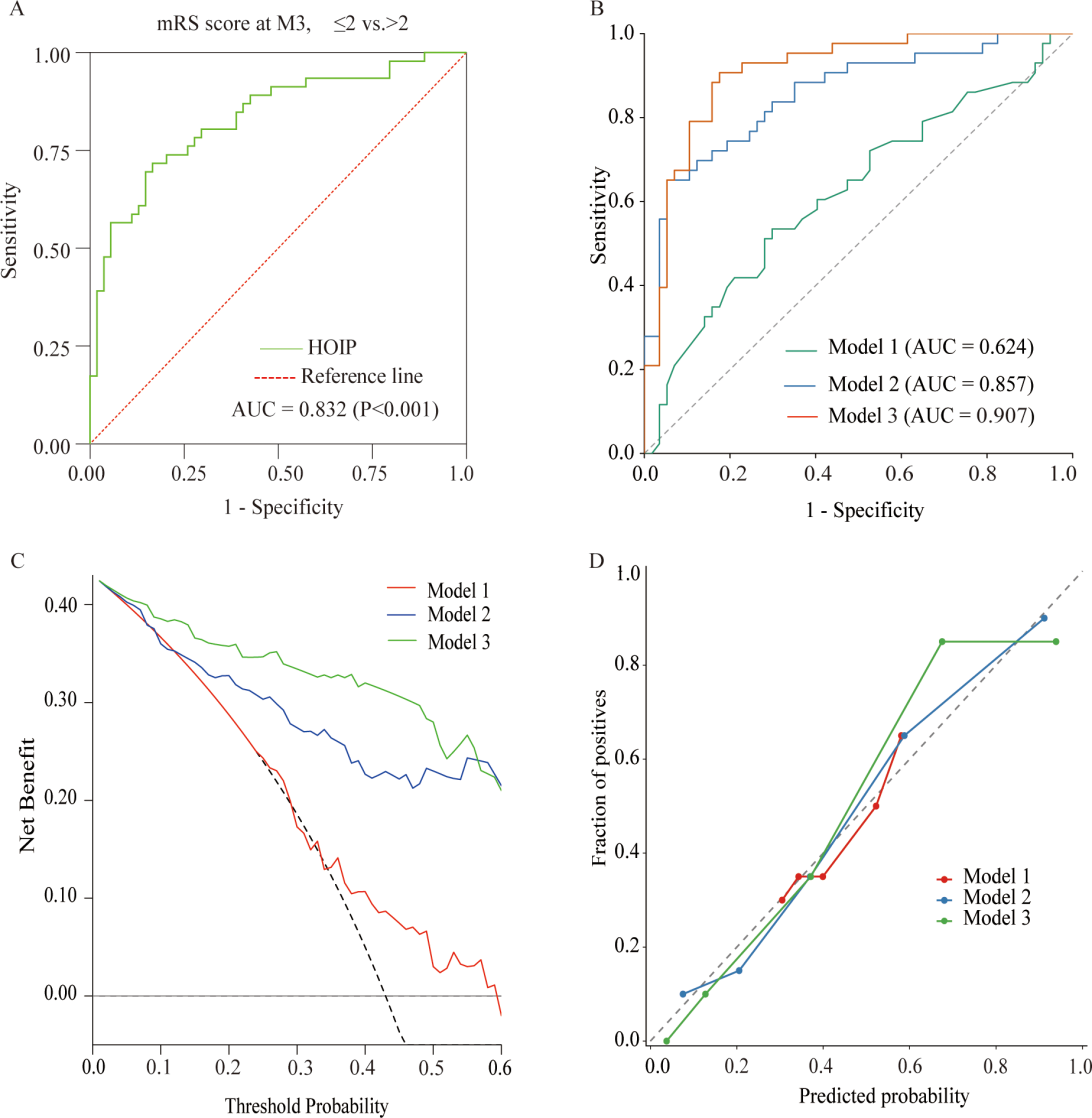
Discrimination, decision curve, and calibration analyses for poor outcome in patients with AIS. **(A)** ROC curves of HOIP for predicting poor functional outcome (mRS >2). **(B)** Comparison of ROC curves for Model 1 (age, sex), Model 2 (Model 1 + NIHSS), and Model 3 (Model 2 + HOIP). **(C)** decision curve analysis of the 3 models. **(D)** calibration plots of the 3 models.

For prediction of functional outcome, HOIP (AUC = 0.832, P < 0.001) showed good discrimination (**Figure 4A**). To further assess the incremental predictive value of HOIP, three prognostic models were constructed. The AUC increased from 0.624 in Model 1 to 0.846 in Model 2, and further to 0.907 in Model 3 (**Figures 4B–D**). Calibration plots showed good agreement between predicted and observed probabilities, and DCA indicated a higher net benefit for Model 3 across a broad range of threshold probabilities. Bootstrap internal validation further supported the robustness of the models, and the detailed results are provided in **Supplementary Table 6**.

### 3.6 Subgroup analysis of HOIP and OTULIN for stroke severity and poor outcome

In subgroup analyses stratified by age, sex, hypertension, diabetes, smoking status, and tertiles of IL-6 and SII, the directions of the associations of HOIP and OTULIN with NIHSS were generally consistent (**Supplementary Tables 7**, **8**). Evidence of effect modification was observed for HOIP across SII tertiles (P for interaction = 0.011), whereas no interaction was detected across the other strata (all P for interaction > 0.05). For OTULIN, no evidence of interaction was observed across any subgroup strata (all P for interaction > 0.05). For poor outcome, no significant effect modification was observed across subgroups defined by age, sex, hypertension, diabetes, or smoking status (all P for interaction > 0.05, **Supplementary Tables 9**, **10**).

## 4 Discussion

This study systematically evaluated the expression characteristics of core components of the linear ubiquitination system, including LUBAC and OTULIN in AIS patients. Peripheral blood HOIP and OTULIN expression was significantly increased in patients with AIS. HOIP expression was positively associated with stroke severity and poor outcome, whereas OTULIN expression was primarily negatively associated with stroke severity. Previous studies suggest that HOIP and OTULIN expression predominantly originates from myeloid cells, including monocytes, macrophages, and neutrophils (40, 41). These cell populations participate in AIS in a temporally distinct manner. Neutrophils are recruited to ischemic brain tissue within minutes to hours after stroke onset and can directly disrupt the BBB and amplify neuroinflammation by releasing reactive oxygen species, matrix metalloproteinases, and neutrophil extracellular traps. Subsequently, monocytes are robustly recruited within 24–48 hours and differentiate into macrophages. T and B cells further contribute to inflammatory regulation approximately 2–3 days after stroke. In addition, the spleen, an important immune reservoir, undergoes contraction during this period and releases stored immune cells into the circulation, particularly monocytes, thereby enhancing immune-cell trafficking to the brain (10). Collectively, the concurrent elevation of peripheral blood HOIP and OTULIN may reflect post-stroke activation of peripheral immune cells and their involvement in inflammatory regulation. Immunofluorescence in autopsy-derived cortical specimens suggested increased expression of LUBAC and OTULIN in the peri-ischemic cortex, providing supportive tissue-level context for the peripheral findings. However, these observations are hypothesis-generating given only two autopsy cases available. Although LUBAC and OTULIN are generally viewed as having opposing roles in inflammatory regulation, their concurrent upregulation in AIS may reflect a more complex response to ischemic injury.

Peripheral blood HOIP was positively correlated with NIHSS, mRS, and IL-6, suggesting that HOIP may reflect the magnitude of inflammatory activation and may be linked to pathways that amplify post-stroke inflammation. As the core catalytic subunit of LUBAC, HOIP stabilizes receptor-associated signaling complexes and augments NF-κB pathway activity through linear ubiquitination of key signaling molecules, including NF-κB essential modulator, inhibitor of NF-κB kinase subunit alpha/beta, tumor necrosis factor receptor 1, TNF receptor–associated factor 2, receptor-interacting serine/threonine-protein kinase 1, and receptor-interacting serine/threonine-protein kinase 3 (42–44). HOIP has also been implicated in macrophage NF-κB activation and M1 polarization, which may exacerbate inflammatory injury (45). During innate immune sensing, HOIP-mediated M1-Ub chain synthesis is required for stimulator of interferon genes-induced NF-κB and interferon regulatory factor 3 activation (46). Excessive NF-κB activation after AIS can intensify immune responses and promote overproduction of proinflammatory cytokines (47, 48). IL-6, a key downstream mediator, can be transcriptionally upregulated by NF-κB and may further amplify inflammatory cascades (49–51). Consistent with prior evidence linking IL-6 to acute immune activation, early neurological deterioration, infarct expansion, and unfavorable outcomes in AIS (50, 52), the observed association between HOIP and IL-6 supports the hypothesis that HOIP upregulation is coupled to heightened inflammatory signaling in the acute phase.

OTULIN is a key negative regulator of inflammation that maintains immune homeostasis by antagonizing LUBAC-mediated M1-Ub chains and restraining excessive NF-κB activation (53, 54). Consistent with this role in peripheral immunity, OTULIN deficiency in human is associated with neutrophilia and recurrent inflammatory episodes, and myeloid cell-specific deletion of OTULIN in mice similarly produces a pronounced inflammatory phenotype (53). Beyond myeloid cells, loss of OTULIN in epithelial tissues alters the composition of the TNFR signaling complex, promotes complex II formation, and induces cell death, thereby increasing susceptibility to inflammation (55). Our previous work showed that ischemic stroke rapidly increases OTULIN expression in the rat brain, and lentivirus-mediated OTULIN overexpression improves neurological function while reducing microglial activation and proinflammatory cytokine release, accompanied by suppression of NF-κB signaling (28). In the present study, peripheral blood OTULIN expression was increased in AIS patients but inversely correlated with stroke severity and IL-6. The inverse association between OTULIN and IL-6 suggests that upregulation of peripheral blood OTULIN may represent an endogenous protective response to ischemic injury. Notably, OTULIN was more closely related to stroke severity than to outcome. Importantly, we observed a significant interaction between HOIP and OTULIN for stroke severity, whereas such an interaction was not evident for outcome. Given that outcome after AIS is determined by multiple factors beyond the initial inflammatory response, such as lesion characteristics, reperfusion status, medical complications, and post-stroke rehabilitation, the absence of a significant interaction for mRS may reflect the greater complexity of prognosis. However, these results remain exploratory and should not be regarded as definitive biomarker validation. Accordingly, the robustness and generalizability of their potential clinical relevance require validation in prospective, large-scale cohorts.

In summary, this study is the first to characterize peripheral blood expression of HOIP and OTULIN during the acute phase of AIS patients and to evaluate their interrelationship. The findings further suggest that HOIP mRNA expression is associated with both stroke severity and functional outcome, whereas OTULIN mRNA expression is primarily associated with stroke severity. Nevertheless, several limitations should be acknowledged. First, the observational associations cannot establish causality. Second, biomarkers were assessed at the mRNA level in peripheral blood. Because peripheral blood samples in this study were collected specifically for mRNA analysis, no matched specimens were preserved for protein-based assay. Future studies should incorporate protein-level confirmation and external validation before these molecules could be considered for translational or clinical application. Third, the autopsy immunofluorescence analysis was based on only two cases. Therefore, these tissue findings should be regarded as supportive rather than confirmatory evidence. Larger autopsy series with quantitative, cell-type–specific analyses will be required to validate and extend these observations. Fourth, HOIP and OTULIN were assessed at a single acute time point, which limited characterization of temporal trajectories and time-dependent analyses. Future longitudinal studies with serial blood sampling will be needed to define the temporal patterns of HOIP and OTULIN expression after stroke. Fifth, although we adjusted for conventional risk factors and inflammatory indicators, residual confounding related to genetic background, metabolic status, or the microbiome cannot be excluded. In addition, baseline imaging burden was not incorporated into the current multivariable models, because imaging measures such as infarct volume, ASPECTS, and pc-ASPECTS were not uniformly available in a standardized and directly comparable form across the entire cohort. Future studies with prospectively standardized imaging protocols will be important to determine whether HOIP and OTULIN provide prognostic information beyond baseline imaging characteristics. Sixth, this study was a single-center observational study with a relatively limited sample size and number of outcome events, which constrained the number of covariates that could be stably incorporated into the multivariable models. Therefore, residual confounding cannot be completely excluded. Seventh, subgroup analyses were underpowered after stratification, and these findings require confirmation in larger cohorts. Eighth, this single-center study did not integrate multidimensional data (e.g., radiomics, transcriptomics, proteomics, or ubiquitinomics), which may limit a comprehensive understanding of the roles of HOIP and OTULIN in AIS. Therefore, multi-center studies with longitudinal follow-up and functional experiments are needed to clarify the mechanistic roles of peripheral blood HOIP and OTULIN in AIS.

## Data Availability

The raw data supporting the conclusions of this article will be made available by the authors, without undue reservation.
