# Supplementary material for: Association of peripheral blood LUBAC and OTULIN expression with severity and outcome in acute ischemic stroke: a prospective cohort study
Source: Front Immunol. 2026 May 20;17:1792936. doi: 10.3389/fimmu.2026.1792936 (PMC13229702; doi:10.3389/fimmu.2026.1792936)
Supplement: Supplementary file 1 [file DataSheet1.docx]

**Supplementary Materials**

**Table S1. Clinical information of the two postmortem brain tissues**

| **Item** | **Patient 1** | **Patient 2** | **Item [Reference range]** | **Patient 1** | **Patient 2** |
| --- | --- | --- | --- | --- | --- |
| Age (y) | 67 | 54 | White blood cell (10^9^ /L) [3.5-9.5] | 18.34 | 11.25 |
| Sex | Female | Female | Monocyte count (10^9^ /L) [0.1-0.6] | 0.99 | 0.51 |
| Smoking | No | No | Neutrophil count (10^9^ /L) [1.8-6.3] | 12.19 | 6.11 |
| Alcohol | No | No | Lymphocyte count (10^9^ /L) [1.3-3.2] | 1.99 | 2.51 |
| BMI (kg/m2 ) | 25.6 | 21.3 | Platelet coun (10^9^ /L) [3.8-5.1] | 4.85 | 4.20 |
| Hyperlipidemia | Yes | Yes | CRP (mg/L) [<5.00] | 78.40 | 6.12 |
| Diabetes mellitus | Yes | Yes | IL-6 (mg/L) [0-4.4] | 13.8 | 7.88 |
| Coronary heart disease | Yes | No | Fasting blood glucose (mmol/L) | 11.3 | 9.01 |
| Atrial fibrillation | No | Yes | Triglycerides (mmol/L) [<1.70] | 1.66 | 3.45 |
| Recurrent stroke | Yes | No | Total cholesterol (mmol/L) [<5.18] | 4.65 | 7.01 |
| Onset-admission time | 16 hour | 6h | LDL-C (mmol/L) [<53.37] | 6.15 | 3.15 |
| TOAST classification | LAA | CE | HDL-C (mmol/L)[1.29-1.55] | 1.18 | 0.77 |
| Relevant treatments | Dehydration therapy for intracranial pressure reduction | Mechanical thrombectomy + anticoagulation | PT (s) [09.8-12.1] | 14.50 | 10.21 |
| key comorbidities | Pulmonary infection, lower-extremity deep venous thrombosis | Pulmonary embolism, pnutritional risk | APTT (s) [23.3-32.5] | 26.3 | 37.8 |
| Stroke onset-to-death interval | 6 days | 8 days | Fibrinogen (g/L) [1.8-3.5] | 4.53 | 7.99 |
| Cause of death | Cerebral herniation | Respiratory failure | D-dimer (ug/mL) [0-1] | 2.13 | 8.04 |
|  |  |  | Homocysteine (umol/L) [<15] | 9.4 | 19.6 |
|  |  |  | NT-proBNP ( pg/ml) [0-125] | 45957.78 | 1360.47 |


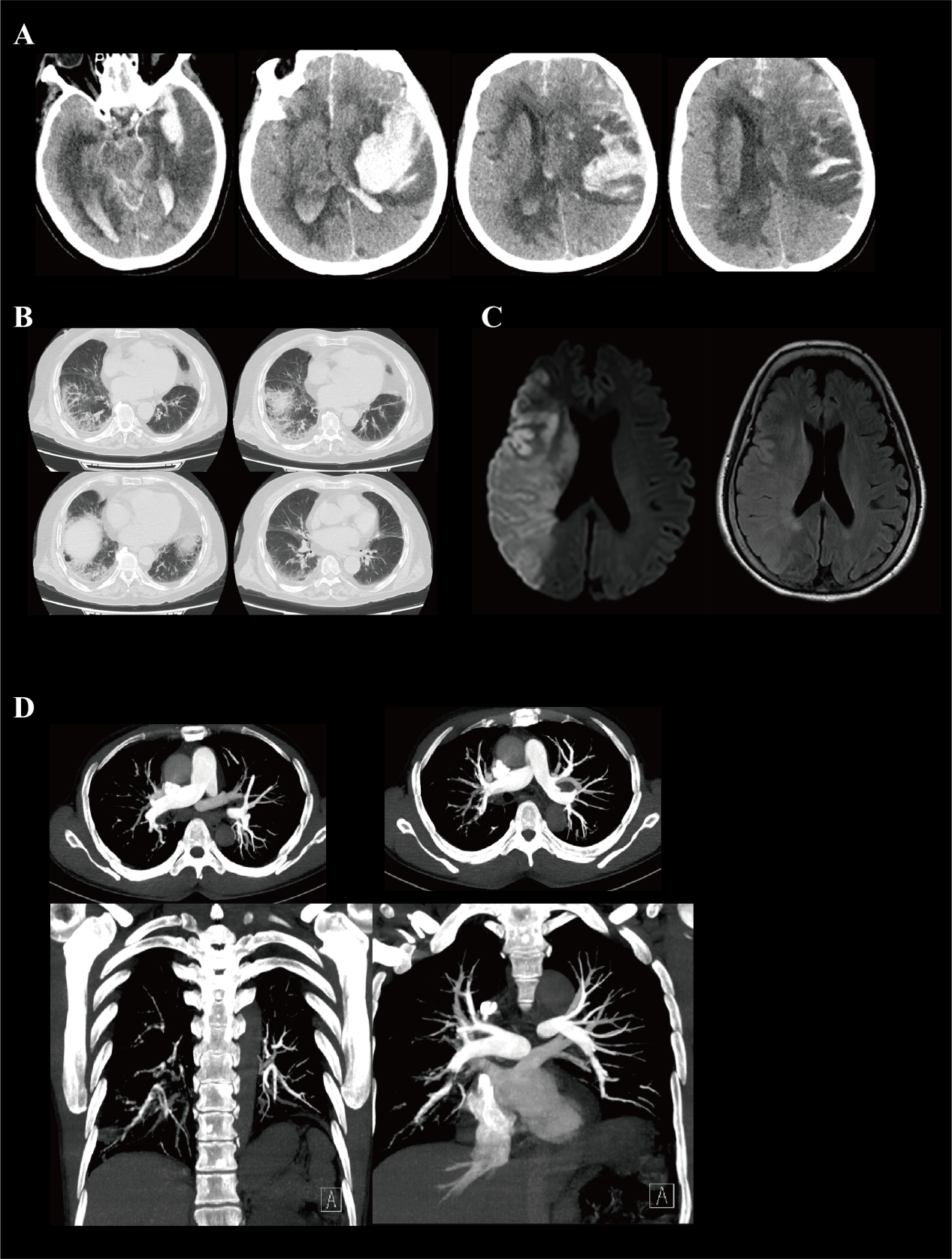


**Supplementary Figure S1. Representative brain and chest imaging findings of the two AIS postmortem cases.** (A) Cranial CT in Patient 1 revealed acute ischemic stroke with hemorrhagic transformation. (B) Chest CT in Patient 1 showed severe pulmonary infection. (C) Diffusion-weighted imaging and T2 Flair cranial MRI in Patient 2 demonstrated acute infarction in the right middle cerebral artery territory. (D) Pulmonary CT angiography in Patient 2 revealed right pulmonary artery embolism with associated thrombus formation.

**Table S2. Ordinal logistic regression analysis of factors associated with NIHSS severity levels**

| Model | Variable | OR (95% CI) | P-value |
| --- | --- | --- | --- |
| Model 1 | HOIP | 2.617 (1.420, 4.823) | 0.002 |
|  | OTULIN | 0.353 (0.229, 0.543) | <0.001 |
|  | Age | 0.990 (0.935, 1.048) | 0.73 |
|  | Male sex | 2.173 (0.730, 6.465) | 0.163 |
| Model 2 | HOIP | 2.686 (1.420, 5.083) | 0.002 |
|  | OTULIN | 0.324 (0.199, 0.526) | <0.001 |
|  | Age | 0.986 (0.929, 1.046) | 0.633 |
|  | Male sex | 2.378 (0.724, 7.816) | 0.154 |
|  | Smoking | 0.865 (0.267, 2.802) | 0.808 |
|  | Hypertension | 0.576 (0.191, 1.739) | 0.328 |
|  | Diabetes | 0.393 (0.132, 1.171) | 0.094 |
| Model 3 | HOIP | 2.878 (1.443, 5.737) | 0.003 |
|  | OTULIN | 0.327 (0.201, 0.531) | <0.001 |
|  | Age | 0.981 (0.922, 1.044) | 0.548 |
|  | Male sex | 2.509 (0.719, 8.751) | 0.149 |
|  | Smoking | 0.730 (0.215, 2.473) | 0.613 |
|  | Hypertension | 0.523 (0.166, 1.652) | 0.269 |
|  | Diabetes | 0.441 (0.145, 1.340) | 0.149 |
|  | CE vs LAA | 0.406 (0.081, 2.043) | 0.274 |
|  | SAA vs LAA | 0.623 (0.160, 2.425) | 0.495 |
|  | SOE/SUE vs LAA | 0.192 (0.028, 1.316) | 0.093 |

Note: The proportional odds assumption was assessed using the Brant test and was not significantly violated in Models 1–3 (Model 1: omnibus P = 0.15; Model 2: omnibus P = 0.47; Model 3: omnibus P = 0.75).

**Table S3. Simple slopes estimates for the OTULIN-NIHSS association at different HOIP quantiles**

| HOIP percentile | Effect of OTULIN on NIHSS (β, 95% CI) | **P value** |
| --- | --- | --- |
| 25% (HOIP = 2.495) | -0.783 (-1.071, -0.495) | <0.001 |
| 50% (HOIP = 3.215) | -1.244 (-1.522, -0.966) | <0.001 |
| 75% (HOIP = 3.787) | -1.611 (-1.948, -1.273) | <0.001 |

Note: β represents the change in NIHSS per unit increase in OTULIN, estimated at the specified HOIP percentile

**Table S4A. Primary and HC3 robust estimates for the interaction model of admission NIHSS score**

| Variable | Primary analysis, β (95% CI) | P value | HC3 robust SE, β (95% CI) | P value |
| --- | --- | --- | --- | --- |
| OTULIN | -1.182 (-1.451, -0.913) | <0.001 | -1.182 (-1.483, -0.880) | <0.001 |
| HOIP | 1.789 (1.206, 2.372) | <0.001 | 1.789 (1.100, 2.478) | <0.001 |
| HOIP × OTULIN | -0.640 (-0.874, -0.406) | <0.001 | -0.640 (-0.940, -0.340) | <0.001 |

Note: The primary analysis was based on the multivariable linear regression interaction model for admission NIHSS score. HC3 robust standard errors were applied to account for heteroscedasticity.

**Table S4B. Sensitivity analyses after excluding influential observations for the interaction model of admission NIHSS score**

| Variable | β (95% CI)  [After excluding influential observations] | P value | β (95% CI)  [After excluding influential observations with HC3 robust SE] | P value |
| --- | --- | --- | --- | --- |
| OTULIN | -1.274 (-1.529, -1.020) | <0.001 | -1.274 (-1.563, -0.985) | <0.001 |
| HOIP | 1.679 (1.147, 2.212) | <0.001 | 1.679 (1.034, 2.324) | <0.001 |
| HOIP × OTULIN | -0.772 (-1.028, -0.516) | <0.001 | -0.772 (-1.078, -0.466) | <0.001 |

Note: Potentially influential observations were identified using Cook’s distance > 4/n and excluded in sensitivity analyses. HC3 robust standard errors were additionally applied after exclusion of influential observations.

**Table S5. Associations of HOIP, OTULIN, and their interaction with admission NIHSS severity categories in ordinal logistic regression models**

| Model | Variable | OR | 95% CI | P value |
| --- | --- | --- | --- | --- |
| Model 1 | OTULIN | 0.284 | (0.172, 0.468) | <0.001 |
|  | HOIP | 2.792 | (1.459, 5.344) | 0.002 |
|  | HOIP × OTULIN | 0.613 | (0.455, 0.826) | 0.001 |
| Model 2 | OTULIN | 0.276 | (0.164, 0.465) | <0.001 |
|  | HOIP | 2.969 | (1.497, 5.887) | 0.002 |
|  | HOIP × OTULIN | 0.598 | (0.445, 0.802) | <0.001 |
| Model 3 | OTULIN | 0.255 | (0.142, 0.457) | <0.001 |
|  | HOIP | 3.604 | (1.551, 8.374) | 0.003 |
|  | HOIP × OTULIN | 0.571 | (0.412, 0.791) | <0.001 |

Note: Model 1 included HOIP, OTULIN, and the HOIP × OTULIN interaction term. Model 2 further adjusted for age and sex. Model 3 further adjusted for smoking, hypertension, diabetes mellitus, and TOAST classification. An OR > 1 indicates higher odds of belonging to a more severe NIHSS category, whereas an OR < 1 indicates lower odds. The proportional odds assumption was assessed using the Brant test and was not violated in Models 1–3 (Model 1: omnibus P = 0.715; Model 2: omnibus P = 0.855; Model 3: omnibus P = 0.992).

**Table S6. Bootstrap internal validation of three prognostic models for poor outcome (mRS > 2)**

| Model | Variables included | Corrected C-statistic | Calibration intercept | Calibration slope | Emax | Corrected Brier score |
| --- | --- | --- | --- | --- | --- | --- |
| Model 1 | Age + sex | 0.593 | -0.038 | 0.8348 | 0.137 | 0.2488 |
| Model 2 | Model 1 + NIHSS score | 0.831 | -0.0317 | 0.9125 | 0.0855 | 0.1685 |
| Model 3 | Model 2 + HOIP | 0.892 | -0.0003 | 0.8576 | 0.0987 | 0.1346 |

Note: Internal validation was performed using 1,000 bootstrap resamples. The corrected C-statistic was derived from the optimism-corrected Somers’ Dxy using the formula: C-statistic = (Dxy + 1) / 2. A calibration intercept closer to 0 and a calibration slope closer to 1 indicate better calibration. Lower Emax and corrected Brier score values indicate lower prediction error and better overall model performance.

**Table S7. Association of HOIP with NIHSS in subgroups**

| Variable | Level | N | β (95% CI) | P value | P for interaction |
| --- | --- | --- | --- | --- | --- |
| Overall |  | 100 | 2.226 (1.465, 2.986) | <0.001 |  |
| Age group |  |  |  |  | 0.082 |
|  | ≤65 years | 55 | 2.658 (1.736, 3.580) | <0.001 |  |
|  | >65 years | 45 | 2.112 (1.049, 3.175) | <0.001 |  |
| Gender |  |  |  |  | 0.281 |
|  | Female | 58 | 1.829 (0.820, 2.838) | <0.001 |  |
|  | Male | 42 | 2.907 (1.518, 4.297) | <0.001 |  |
| Hypertension |  |  |  |  | 0.383 |
|  | No | 60 | 2.336 (1.310, 3.362) | <0.001 |  |
|  | Yes | 40 | 1.832 (0.621, 3.043) | 0.003 |  |
| Diabetes |  |  |  |  | 0.404 |
|  | No | 61 | 2.397 (1.481, 3.313) | <0.001 |  |
|  | Yes | 39 | 1.595 (0.142, 3.049) | 0.031 |  |
| Smoking |  |  |  |  | 0.127 |
|  | No | 52 | 2.993 (1.784, 4.201) | <0.001 |  |
|  | Yes | 48 | 1.773 (0.686, 2.860) | 0.001 |  |
| IL-6 tertiles |  |  |  |  | 0.557 |
|  | T1 (Low) | 34 | 0.594 (-0.511, 1.700) | 0.292 |  |
|  | T2 (Medium) | 33 | 1.525 (0.198, 2.851) | 0.024 |  |
|  | T3 (High) | 33 | 3.804 (2.147, 5.462) | <0.001 |  |
| SII tertiles |  |  |  |  | 0.011 |
|  | T1 (Low) | 34 | 0.873 (-0.354, 2.101) | 0.163 |  |
|  | T2 (Medium) | 33 | 2.992 (1.544, 4.441) | <0.001 |  |
|  | T3 (High) | 33 | 1.762 (0.082, 3.441) | 0.04 |  |

**Table S8. Association of OTULIN with NIHSS in subgroups**

| Variable | Level | N | β (95% CI) | P value | P for interaction |
| --- | --- | --- | --- | --- | --- |
| Overall |  | 100 | -2.500 (-3.312, -1.688) | <0.001 |  |
| Age group |  |  |  |  | 0.425 |
|  | ≤65 years | 55 | -2.150 (-3.955, -0.345) | 0.02 |  |
|  | >65 years | 45 | -2.448 (-3.362, -1.534) | <0.001 |  |
| Gender |  |  |  |  | 0.731 |
|  | Female | 58 | -2.560 (-3.491, -1.629) | <0.001 |  |
|  | Male | 42 | -2.912 (-4.488, -1.337) | <0.001 |  |
| Hypertension |  |  |  |  | 0.208 |
|  | No | 60 | -2.506 (-3.694, -1.319) | <0.001 |  |
|  | Yes | 40 | -2.204 (-3.278, -1.131) | <0.001 |  |
| Diabetes |  |  |  |  | 0.861 |
|  | No | 61 | -2.611 (-3.677, -1.546) | <0.001 |  |
|  | Yes | 39 | -2.142 (-3.510, -0.774) | 0.002 |  |
| Smoking |  |  |  |  | 0.681 |
|  | No | 52 | -2.370 (-3.420, -1.320) | <0.001 |  |
|  | Yes | 48 | -2.527 (-3.834, -1.219) | <0.001 |  |
| IL-6 tertiles |  |  |  |  | 0.207 |
|  | T1 (Low) | 34 | -1.425 (-2.063, -0.788) | <0.001 |  |
|  | T2 (Medium) | 33 | -2.741 (-4.332, -1.151) | <0.001 |  |
|  | T3 (High) | 33 | -4.324 (-6.509, -2.139) | <0.001 |  |
| SII tertiles |  |  |  |  | 0.133 |
|  | T1 (Low) | 34 | -2.028 (-3.083, -0.974) | <0.001 |  |
|  | T2 (Medium) | 33 | -2.751 (-4.072, -1.430) | <0.001 |  |
|  | T3 (High) | 33 | -2.636 (-5.078, -0.194) | 0.034 |  |

**Table S9. Association of HOIP with poor outcome (mRS > 2) in subgroups**

| Subgroup | Level | N | OR (95% CI) | P value | P for interaction |
| --- | --- | --- | --- | --- | --- |
| Overall |  | 100 | 4.590 (2.420, 8.710) | <0.001 |  |
| Age group |  |  |  |  | 0.932 |
|  | ≤65 years | 55 | 4.720 (2.030, 10.990) | <0.001 |  |
|  | >65 years | 45 | 4.460 (1.650, 12.060) | 0.003 |  |
| Gender |  |  |  |  | 0.641 |
|  | Female | 58 | 5.290 (2.110, 13.230) | <0.001 |  |
|  | Male | 42 | 3.880 (1.530, 9.830) | 0.004 |  |
| Hypertension |  |  |  |  | 0.652 |
|  | No | 60 | 5.170 (2.220, 12.030) | <0.001 |  |
|  | Yes | 40 | 3.830 (1.430, 10.260) | 0.007 |  |
| Diabetes |  |  |  |  | 0.743 |
|  | No | 61 | 4.460 (2.060, 9.660) | <0.001 |  |
|  | Yes | 39 | 5.730 (1.580, 20.880) | 0.008 |  |
| Smoking |  |  |  |  | 0.522 |
|  | No | 52 | 6.400 (2.250, 18.250) | 0.001 |  |
|  | Yes | 48 | 4.090 (1.680, 9.930) | 0.002 |  |
| IL-6 tertiles |  |  |  |  | 0.047 |
|  | T1 (Low) | 34 | 2.840 (0.790, 10.250) | 0.11 |  |
|  | T2 (Medium) | 33 | 2.620 (1.120, 6.170) | 0.027 |  |
|  | T3 (High) | 33 | 50.660 (1.740, 70.150) | 0.022 |  |
| SII tertiles |  |  |  |  | 0.220 |
|  | T1 (Low) | 34 | 9.380 (1.710, 51.320) | 0.01 |  |
|  | T2 (Medium) | 33 | 7.210 (1.830, 28.370) | 0.005 |  |
|  | T3 (High) | 33 | 2.490 (1.080, 5.740) | 0.033 |  |

**Table S10. Association of OTULIN with poor outcome (mRS > 2) in subgroups**

| Subgroup | Level | N | OR (95% CI) | P value | P for interaction |
| --- | --- | --- | --- | --- | --- |
| Overall |  | 100 | 0.420 (0.300, 0.580) | <0.001 |  |
| Age group |  |  |  |  | 0.865 |
|  | ≤65 years | 55 | 0.420 (0.270, 0.660) | <0.001 |  |
|  | >65 years | 45 | 0.400 (0.240, 0.680) | 0.001 |  |
| Gender |  |  |  |  | 0.278 |
|  | Female | 58 | 0.360 (0.220, 0.580) | <0.001 |  |
|  | Male | 42 | 0.520 (0.330, 0.840) | 0.007 |  |
| Hypertension |  |  |  |  | 0.465 |
|  | No | 60 | 0.450 (0.300, 0.670) | <0.001 |  |
|  | Yes | 40 | 0.340 (0.180, 0.650) | 0.001 |  |
| Diabetes |  |  |  |  | 0.329 |
|  | No | 61 | 0.470 (0.320, 0.700) | <0.001 |  |
|  | Yes | 39 | 0.330 (0.180, 0.600) | <0.001 |  |
| Smoking |  |  |  |  | 0.980 |
|  | No | 52 | 0.420 (0.270, 0.660) | <0.001 |  |
|  | Yes | 48 | 0.420 (0.260, 0.700) | 0.001 |  |
| IL-6 tertiles |  |  |  |  | 0.396 |
|  | T1 (Low) | 34 | 0.590 (0.370, 0.950) | 0.029 |  |
|  | T2 (Medium) | 33 | 0.380 (0.190, 0.740) | 0.004 |  |
|  | T3 (High) | 33 | 0.360 (0.180, 0.740) | 0.005 |  |
| SII tertiles |  |  |  |  | 0.423 |
|  | T1 (Low) | 34 | 0.220 (0.070, 0.640) | 0.006 |  |
|  | T2 (Medium) | 33 | 0.400 (0.210, 0.750) | 0.004 |  |
|  | T3 (High) | 33 | 0.460 (0.260, 0.810) | 0.007 |  |
